# Supplementary material for: Netcombin: An algorithm for constructing optimal phylogenetic network from rooted triplets
Source: PLoS One. 2020 Sep 18;15(9):e0227842. doi: 10.1371/journal.pone.0227842 (PMC7500971; doi:10.1371/journal.pone.0227842)
Supplement: S4 File — (ZIP) [file pone.0227842.s004.zip › SequenceData-Triplet/sequenceData-10-20-40-Triplet2/data-(sample 10- length 500- num seq 40- m rate 10^-6)/1 seq.pdf]

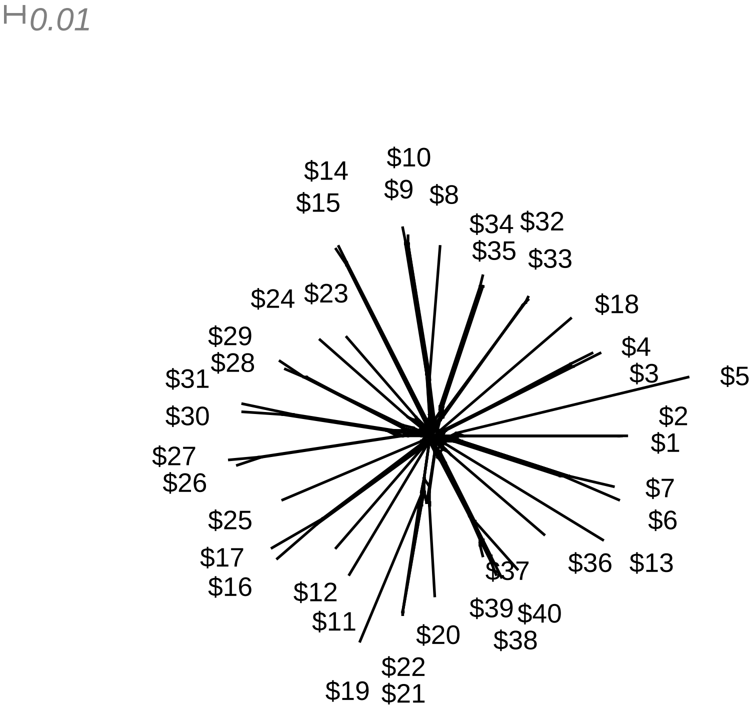
0.01
$10
$14
$9
$8
$15
$34
$32
$35
$33
$24
$23
$18
$29
$4
$28
$3
$31
$5
$30
$2
$1
$27
$26
$7
$6
$25
$17
$36
$13
$16
$37
$12
$39
$40
$11
$20
$38
$22
$19
$21
